# Flow photochemistry: Old light through new windows

**DOI:** 10.3762/bjoc.8.229

**Published:** 2012-11-21

**Authors:** Jonathan P Knowles, Luke D Elliott, Kevin I Booker-Milburn

**Affiliations:** 1 School of Chemistry, University of Bristol, Cantock’s Close, Bristol, BS8 1TS, UK.

**Keywords:** cycloaddition, flow chemistry, photocatalysis, photochemistry, photooxygenation

## Abstract

Synthetic photochemistry carried out in classic batch reactors has, for over half a century, proved to be a powerful but under-utilised technique in general organic synthesis. Recent developments in flow photochemistry have the potential to allow this technique to be applied in a more mainstream setting. This review highlights the use of flow reactors in organic photochemistry, allowing a comparison of the various reactor types to be made.

## Introduction

The use of ultraviolet light to carry out bond-forming reactions in synthetic organic chemistry has a long history dating back to the mid-19th century. The observation by Trommsdorff [1] that crystals of the sesquiterpene santonin would literally burst open upon exposure to sunlight can perhaps be considered as the beginning of organic photochemistry. In 1883 Cannizzaro and Sestini [2] investigated this further and reported the formation of photosantonic acid upon irradiation of santonin. It is generally regarded that the systematic and ground-breaking investigations of Ciamician and Silber [3] paved the way for modern synthetic photochemistry. At the turn of the 20th century they described the first examples of now common reactions such as intramolecular [2 + 2] cycloaddition; basic ketone photochemistry such as α- and β-cleavage; as well as fundamental concepts such as the singlet and triplet states and *n*,π* and π,π* excited states. From the late 1950s onwards thousands of examples of the application of photochemistry in synthesis were reported. Eaton's cubane synthesis [4], Corey's synthesis of carophyllene alcohol [5–6] and Wender's synthesis of cedrene [7] are just three outstanding examples to highlight. Photochemistry has also made the transition to industrial-scale synthesis. For example the Toray process [8–10] for the synthesis of caprolactam, used to manufacture Nylon 6, proceeds by irradiation of cyclohexane with NOCl and HCl, and is carried out in dedicated plants producing >100,000 tons per annum.

### Conventional techniques & equipment

**Figure 1:**
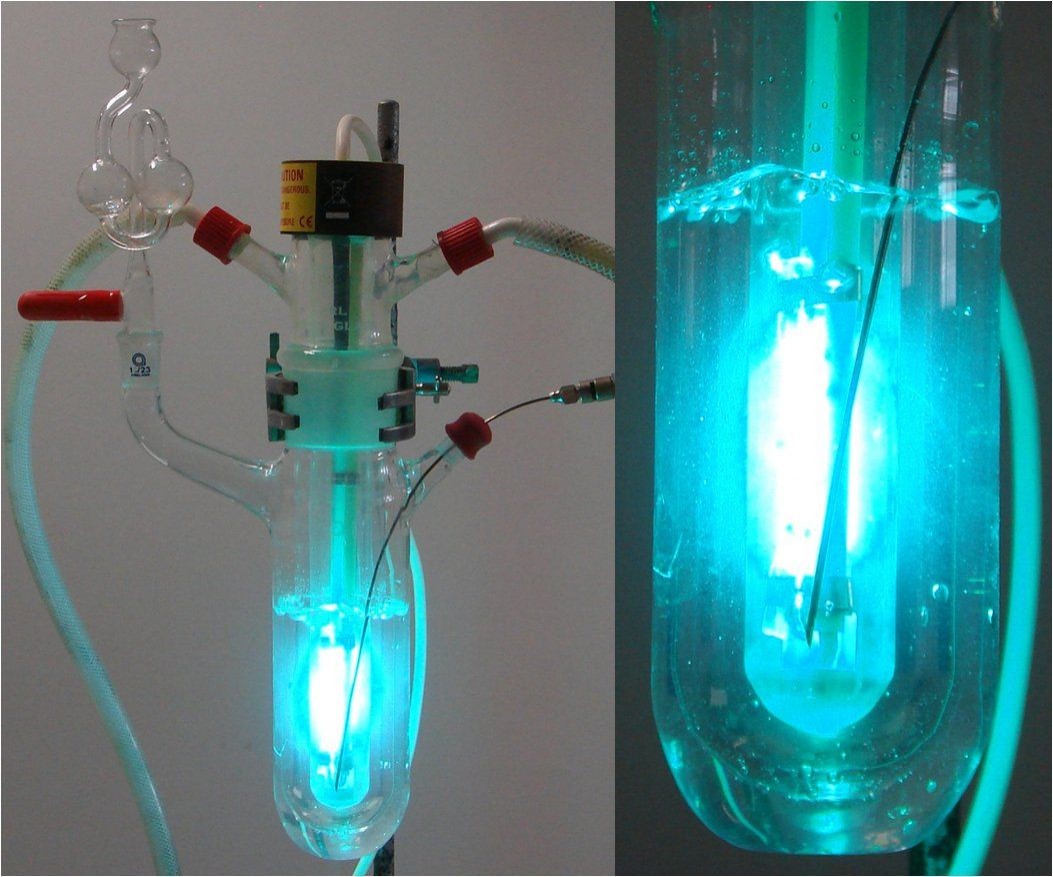
An immersion-well batch reactor with 125 W medium pressure Hg lamp.

For well over half a century the most dependable apparatus for laboratory scale organic photochemistry has been the immersion-well photoreactor in conjunction with mercury-vapour-discharge lamps (Figure 1). This compact batch reactor is an excellent device to carry out preparative photochemistry on scales of milligrams up to a few grams. The lamp is contained in a double-jacketed water-cooled immersion well. This is then placed into a reaction flask containing the chromophoric substrate. This flask is usually standard Pyrex glassware. The solution is normally degassed to remove oxygen in order to diminish the possibility of quenching and other reactions, such as peroxide formation (conveniently achieved with a long needle and nitrogen stream). For safety, the cooling water should be connected to a flow sensor in order to shut down the lamp should the water pressure drop. The whole apparatus can be shielded in a cabinet to avoid exposure to powerful UV radiation. Alternatively aluminium foil can be wrapped around the glassware to achieve a very effective level of shielding. UV goggles/visor should be worn if unshielded apparatus is operated, and especially during sampling of the reaction mixture. Once all this is in place the lamp is then switched on and the progress of the photochemical reaction can be monitored by conventional means (TLC, GC, LCMS). As often no reagents are used, workup is by simple evaporation of solvent, and the product is purified by conventional means.

The most common UV sources are the commercially available mercury-discharge lamps. These are evacuated glass tubes containing mercury vapour through which an electrical discharge passes. This results in the excitation of the Hg atoms and a subsequent emission of UV radiation. The two most common Hg lamps are

• Low pressure: These are similar to everyday fluorescent lamps, and input powers range from 6 up to 300 W and above. However, the latter are generally very large (1–2 metres in length) and not suited to general laboratory use. Lamps in the range 6–16 W are low-priced devices and are generally very efficient in their conversion of input power into UV (30%). Uncoated lamps emit the bulk (90%) of their spectral output at 254 nm (UVC) and are particularly suited to carbonyl and arene photochemistry as well as halogenation chemistry. These lamps are also available with a range of phosphor coatings to emit both UVB and UVA radiation. They have found commercial use in medical, tanning and insect-attraction applications. These lamps have very long lifetimes, often in excess of several thousand hours.

• Medium pressure: These are much higher power lamps of input powers ranging from 125 W up to large 60 kW lamps for industrial purposes (e.g., the Toray process). In standard laboratory use, lamps of 125 and 400 W are the most common. These lamps are broadband emitters with the most powerful UV output in the 300–370 nm region. Strong emissions in the IR region account partly for their high operating temperatures meaning they must be used in an appropriate water-cooled immersion-well apparatus. They usually have reliable lifetimes of a few hundred hours, which can be extended considerably if left on. They can be used for general-purpose photochemistry and are particularly suited for chromophores absorbing strongly in the 290–400 nm region.

The glassware used for the immersion-well is particularly important as it functions as a useful filter with medium pressure lamps: quartz is essentially transparent from 200 nm to visible; Vycor >240 nm; Pyrex >300 nm; uranium glass >350 nm. It should be noted that Vycor and uranium glass are now difficult to source due to manufacturing issues. Often it is more convenient to purchase a quartz immersion well and use a glass filter, e.g., a tube of Pyrex can be placed between the lamp and the inner wall of the immersion well, thus filtering out radiation below 300 nm.

Finally, solvent choice is also a key factor. The solvent must be able to dissolve a range of different substrates but must not be a strong UV absorber itself. Other factors to consider are that the solvent should not undergo quenching or hydrogen-atom abstraction or other reactions with the excited state (although some solvents can be useful sensitisers, e.g., acetone). Acetonitrile has proved to be a particularly versatile solvent as it is economical, good at dissolving polar substrates, does not absorb above 200 nm, and is easy to remove on a rotary evaporator.

Other reactor systems have been developed over the years and include

• Multiple lamp or “Rayonet” reactors. This is a cabinet where multiple lamps direct their radiation towards a sample at the centre. There is usually a fan located at the bottom of the cabinet to ensure sufficient cooling. These are useful for scaling up batch reactions that use low-pressure lamps.

• Falling-film reactors. A falling-film reactor is particularly useful for scaling up the photolysis of a strongly absorbing chromophore. Here, a thin film of the substrate solution flows down a glass plate or tube in close proximity to the light source. The short path length leads to very efficient irradiation. The downside is that residence time is short and often the solution has to be recirculated, leading to the possibility of side reactions. Nonetheless, this has proved to be a valuable device in the right circumstances. For example, Griesbeck [11] reported the design of a particularly useful version in conjunction with a high power 308 nm XeCl excimer source.

Why then, with this wealth of useful reactions and established techniques at hand, do mainstream organic chemists tend to avoid photochemistry as a routine synthetic tool?

There are a number of likely contributing factors:

• Equipment. The first time user is often confronted by a lack of suitable equipment or know-how, e.g., which lamp, glassware and solvent to use.

• Safety. Medium pressure mercury lamps operate optimally at ~600 °C and emit intense and potentially damaging UV radiation.

• Difficulty in scaling up. The Toray caprolactam process proves this is not a problem at the industrial scale. However, in the lab it is often very difficult to scale up above a few grams in a classic immersion-well reactor (see below).

It is perhaps then not surprising that synthetic chemists, as potential first-time users, avoid this medium. This in turn leads to the more fundamental problem: synthetic chemists do not generally think *photo-retrosynthetically.* As a result potentially shorter and more efficient synthetic routes to complex organic molecules, as well as access to new molecular space have long been avoided by mainstream synthetic chemists.

### Flow to the rescue?

Over the last 15 years flow chemistry has begun to make a major impact in the way many organic chemists perform synthesis. The pioneering work of Ley [12–14] and others [15] has demonstrated that complex organic molecules can be constructed continuously in well-designed multireactor systems linked in sequence and under precise software control. Nearly all common batch reactions [16] can now be carried out in flow.

One of the key issues of scaling-up organic photochemistry in an immersion-well (batch) reactor is that light penetration to the surrounding solution is limited by the high absorption of the substrate and falls off rapidly with distance from the lamp. This effect is best explained by considering a few basic equations. The absorption of light by a solution (*A*) shows a linear relationship with the extinction coefficient (ε), the molar concentration (*c*) and the path length (*I*) as described by the Beer–Lambert law (Equation 1). The absorption, however, is expressed as a logarithmic function of the ratio of transmitted light (*I*) to incident light (*I*_0_) (Equation 2).

[1]
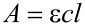


[2]
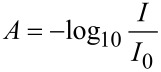


**Figure 2:**
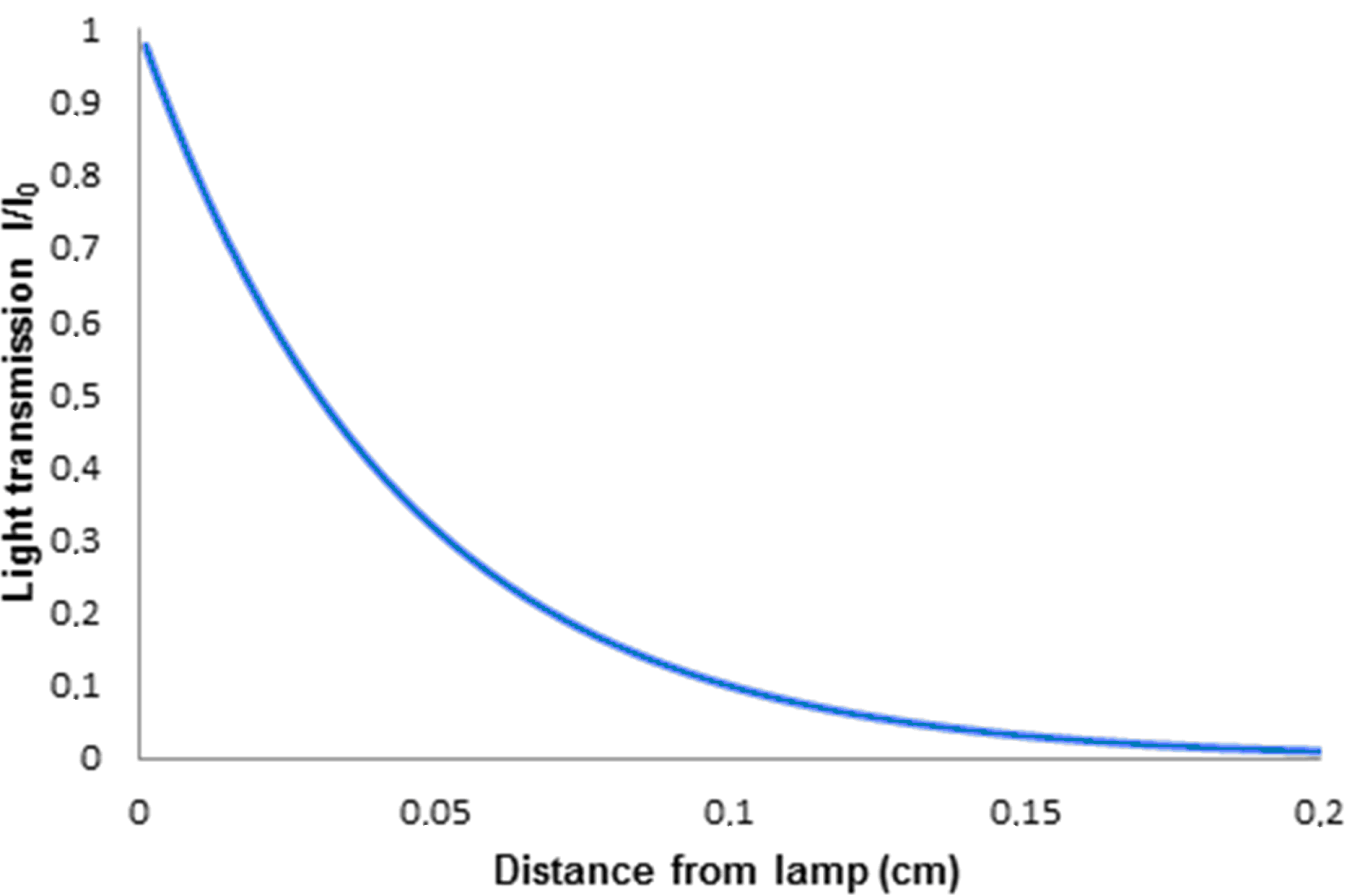
Transmission profile of a 0.05 M solution, ε = 200 M^−1^ cm^−1^.

An absorption of 1 therefore represents a situation where 90% of light is absorbed (*I* = 0.1 *I*_0_). For example, a weak π, π* absorption such as the forbidden band of benzene at 254 nm has an extinction coefficient of about 200 M^−1^ cm^−1^. The path length required for a solution of a modest concentration of 0.05 M to absorb 90% of the incident light will be just 0.1 cm, or 1 mm. The transmission profile for such a solution is shown in Figure 2.

A more typical π, π* transition may have an extinction coefficient of about 20,000 M^−1^cm^−1^ and in this case a 0.05 M solution will absorb 90% if incident light at a distance of 0.01 mm.

Essentially the reaction solution (photolysate) nearest the lamp “screens” the bulk of the reaction solution from UV. This effect is also amplified if the reaction solution is concentrated. When the scale of the reaction is increased with the same lamp it becomes increasingly more difficult to drive the reaction to completion. Attempts to do so often result in the “curse” of synthetic photochemistry, i.e., over-irradiation of the product and formation of side products and photopolymers.

This is where flow photochemistry becomes a very attractive proposition, as in principle it can overcome all the key problems of batch photochemistry in the laboratory.

• At any one time under flow conditions only a very small amount of the total reaction solution “sees” intense UV irradiation from the UV source. This leads to very efficient, uniform irradiation of the whole reaction solution over time.

• The UV exposure time can be precisely controlled by the flow-rate and reactor volume. This can address both the under- and over-irradiation problems encountered with batch reactors.

• As a *continuous* flow device is scale independent, a single reactor can in principle be used to process a few milligrams of substrate up to nearly a kilogram per day (see macroreactors).

• Due to much shorter path lengths high concentration solutions can be irradiated effectively.

• Large volumes of very low concentration solutions can be irradiated. This is particularly useful for reactions with competing intermolecular side reactions, e.g., dimerisation and polymerisation.

• The photolysate can be concentrated by continuous evaporation and the solvent recycled with the starting material. This can dramatically cut down the solvent footprint, particularly in dilute reactions where large volumes of solvent would be required to process quantities of substrate.

• Safety. By allowing the bulk solution to be kept remote from the lamp, only a minimal amount of flammable solvent is near a potential ignition source at any one time.

Historically, there have been a few reports of the applications of rudimentary flow techniques in photochemistry, such as the use of a spiral glass reactor in vitamin D synthesis (1959) [17] and the use of coiled teflon tubing as a gas-phase reactor for the synthesis of methyl chloride (1971) [18]. However, it was not until the turn of the 21st century that the application of flow devices to synthetic photochemistry really started to grow. For the purposes of this review, there are broadly speaking two types of flow-reactor system that have been used for synthetic photochemistry.

**Figure 3:**
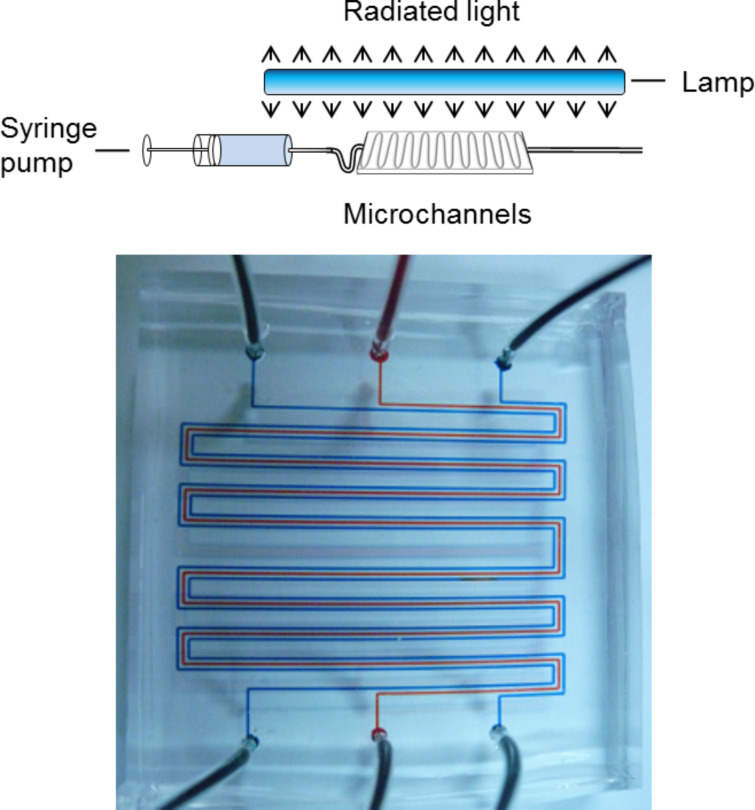
Schematic of a typical microflow photochemical reactor (above) and detail of a triple-channel microflow reactor (below) used for the photooxygenation of citronellol [20].

**Figure 4:**
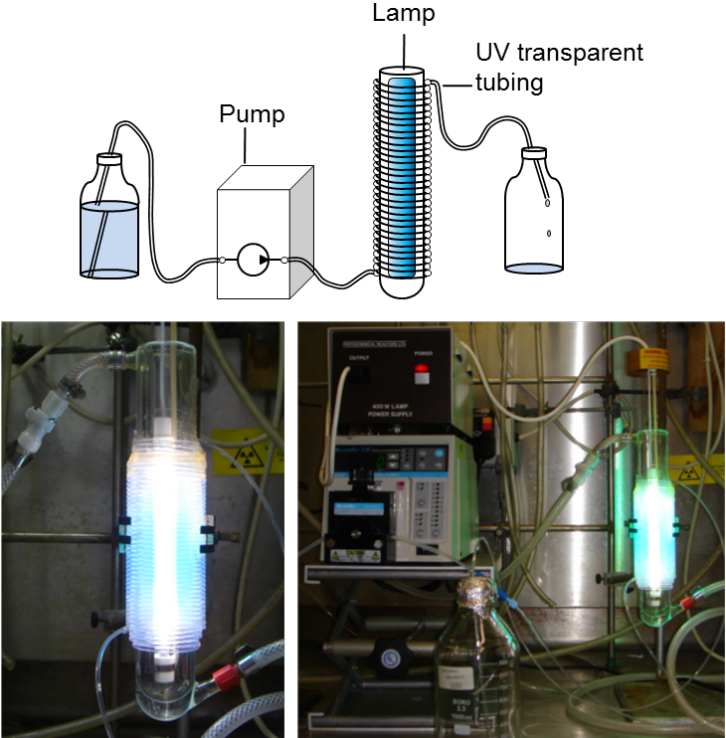
Schematic of a typical macroflow photochemical reactor (above) and images of the FEP photochemical flow reactor developed by Booker-Milburn and Berry [21].

• Microflow [19]. These devices consist of fabricated microchannels and range from bespoke “lab-on-a-chip” designs to highly engineered glass and metal systems. They are generally defined as having channels less than 1 mm in thickness and typical throughput flow rates range from a few microliters up to 1 mL per minute. A syringe pump is ideally suited to delivering solutions to these reactors (Figure 3).

• Macroflow. These devices generally involve UV-transparent tubing (>0.5 mm, i.d.) wrapped around a high-power UV source and have flow rates usually greater than 1 mL/min (Figure 4).

The primary purpose of any photochemical reactor is to allow a solution to be irradiated by the emissions from a light source in a controlled manner. It is useful to consider a lamp as emitting a flux of photons. An equation to calculate the number of moles of photons (einsteins) per hour at a given wavelength (λ) if the total power of emissions at that wavelength is known, is shown in Equation 3.

[3]
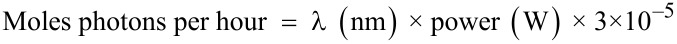


This equation is particularly useful when considering selective narrowband emitters such as a low-pressure mercury lamp or LEDs. For example, a 15 W low-pressure lamp, if operating at 30% efficiency will have a total UV power of 4.5 W at 254 nm. According to Equation 3 this corresponds to a photon flux of 34 mmol photons per hour. When multiplied by the quantum yield of a reaction driven by 254 nm UV, a figure is obtained that represents the maximum theoretical productivity of the reaction *if all the emitted photons are absorbed.*

The small aperture and limited channel coverage of a microflow reactor puts it at a distinct disadvantage in this aspect. The microchannels are milled or etched into a *planar* surface and cannot efficiently capture the *radial* emission from most common light sources. Even LEDs, which can be considered as planar light sources, often have a beam width wider than the actual channels; however, this issue could easily be addressed by a more directed reactor design. As a result microflow reactors are often inefficient at capturing light and suffer poor productivity.

The main advantage at present of the microflow photochemical reactors is the exquisite control over reaction conditions they can offer. The precisely engineered channels can be made shallow enough to ensure uniform irradiation of concentrated or strongly absorbing solutions. Temperature control is also more effective than in larger systems offering the possibility of studying photochemistry outside the normal range. When coupled with online analysis, these reactors can potentially enable the rapid screening of reactions and conditions for optimisation and discovery.

The most efficient method of capturing the maximum number of photons, and hence to maximise productivity, is to construct the reactor *around* the lamp. This approach has been met with great success in the field of macroflow photochemistry. These reactors can easily be constructed, even by a novice, using cheap, readily available materials. Essentially, all that is required is some UV-transparent tubing, a pump and a lamp. A water cooled jacket is not even required if a low pressure lamp is used, due to the mild operating temperatures (40 °C).

Such a reactor can make efficient use of the 15 W lamp described earlier. For example, when 80% of the available photons are delivered to the solution to drive a reaction with a quantum yield of just 10%, the productivity can be estimated as 2.7 mmol/h. The photons are acting as a “reagent” but not one that can be added all at once; rather, they are introduced as a stream (flux). As the reactant solution is also introduced as a stream, the flow rate can be precisely tailored to match the photon flux of the lamp and the quantum yield of the substrate.

The main aim of this review is to illustrate the advances made in flow photochemistry over the last 10 years. This also presented an opportunity to compare different reactor types in areas such as selectivity, yield and productivity [22].

## Review

### Microflow photochemistry

#### Photocycloadditions

**Scheme 1:**
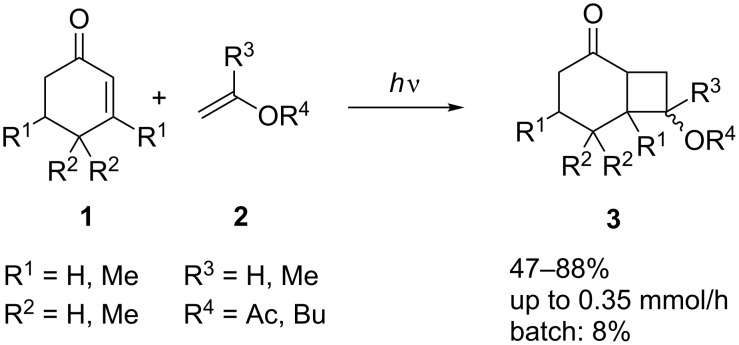
[2 + 2] photocycloadditions of enones with enol derivatives.

Although many photocycloadditions have been performed by using other types of reactor, only [2 + 2] cycloadditions have been performed in microflow systems. The first of these was an intermolecular reaction between enones and vinyl esters or ethers (Scheme 1). By using a 300 W Hg lamp and a FOTURAN glass reactor the reaction gave moderate to high yields, albeit with poor diastereoselectivity [23]. Subsequent work demonstrated that the photon efficiency of the reaction could be improved through the use of a 15 W black light and a quartz-plate-covered microreactor [24]. Although faster reaction times are claimed for the microflow system as compared to batch, it is probably unrealistic to compare reaction completion times in two reactors whose volumes are so different. Material output per unit time provides a better comparison, and in this case shows the batch reactor to be uncompetitive when compared to two microflow reactors connected in series (0.35 mmol/h in flow versus 0.02 mmol/h in batch) [23].

**Scheme 2:**
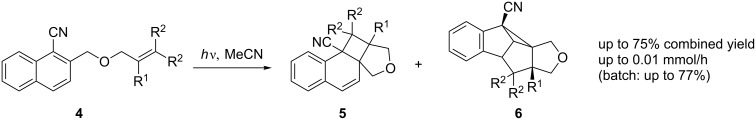
Competing reactions in an intramolecular [2 + 2] photocycloaddition.

Intramolecular [2 + 2] photocycloadditions have also been performed by using microflow apparatus. Mizuno et al. reported the reaction shown in Scheme 2 using a Xe lamp (λ > 290 nm) in a poly(dimethylsiloxane) reactor [25]. The microreactor was compared with a batch reactor and was found to give slightly better selectivity (59:9 versus 56:17) for 4,5-fused system **5** over a 4,6-fused system **6**. However, conversion in the microflow system was lower than that in the batch reactor [25]. This was later shown to be due to the reversibility of the cycloaddition to yield **5**, whilst the reaction to yield **6** is irreversible. Thus, attempts to achieve high conversions always increased the proportion of product **6** [26]. Use of the microreactor proved to be an advantage in this situation as compound **5** was removed as it was formed and was not exposed to further irradiation. In this way high selectivity (96:4) could be achieved in flow whilst little selectivity (55:45) was achieved in batch for the same conversion. Increasing the width of the channel in the flow reactor allowed flow rates to be increased whilst retaining this level of selectivity; however, productivity even with this larger reactor was still extremely low (0.014 mmol/h) [26].

**Scheme 3:**
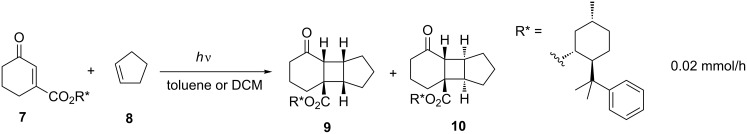
Diastereocontrolled cycloaddition of a cyclic enone with cyclopentene.

Asymmetric induction can be achieved in [2 + 2] cycloadditions through the use of a chiral auxilliary. The [2 + 2] reaction shown in Scheme 3 employed a chiral ester function to direct the facial selectivity of the addition of the enone to cyclopentene. Diastereoselectivity was found to be largely independent of solvent (DCM versus toluene) but was dependent on temperature. Thus, cooling the reaction from 0 °C to −40 °C gave an increase in d.e. of compound **9** from 71% to 82%; this also affected the selectivity of **9** versus **10**, which changed from 39:61 to 1:1 under the same conditions. Interestingly, the microflow reactor gave better diastereoselectivity for compound **9** than a batch reactor under the same conditions (82% d.e. versus 72% d.e.). This effect was ascribed to the more efficient temperature control due to the smaller reactor volume. However, at 0.02 mmol/h the productivity of the microreactor was lower than would be expected of a batch reactor [27]. A similar, non-diastereoselective version of this reaction has been performed by using a medium-pressure Hg lamp [28].

**Scheme 4:**
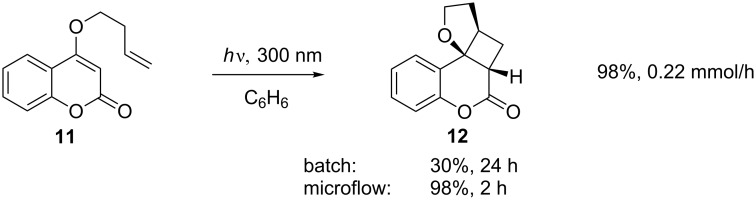
Comparison of yields and reaction times for a batch reactor with a microflow system.

Another [2 + 2] reaction has been performed in the LOPHTOR stainless steel channel reactor. The cycloaddition shown in Scheme 4 was performed in this reactor and compared to the same reaction performed in a conventional batch reactor. Despite the increased yield and decreased irradiation time, achieving a real comparison of the two reactor outputs is difficult given the lack of details regarding the batch process. The highest productivity from the microflow system was 0.22 mmol/h. It is hard to give a meaningful comparison with the batch reaction as no scale is reported; however, it is possible to state that the batch reaction would have had to be performed on a 17.6 mmol scale in the given time period to be competitive [29].

**Scheme 5:**
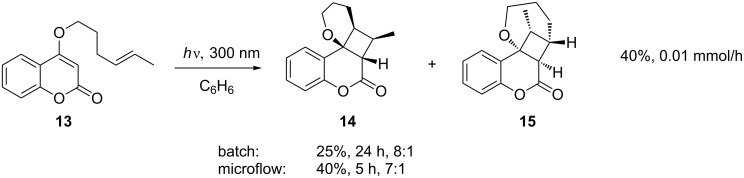
Intramolecular [2 + 2] photocycloaddition.

In a similar [2 + 2] photocycloaddition (Scheme 5), the authors demonstrated that by extending the chain length by one carbon, the 4,6-fused ring system **14** could be formed, albeit with some formation of regioisomer **15**. In this instance the batch reaction gave slightly better selectivity than that seen in the microflow system [29].

**Scheme 6:**
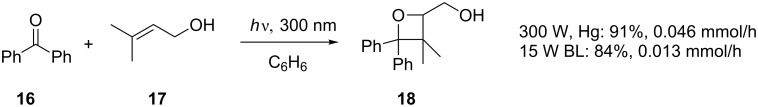
Paterno–Büchi reaction of benzophenone with an allylic alcohol.

The Paterno–Büchi reaction has also been explored in a microflow setting (Scheme 6). A 15 W black light was again found to be a more photon-efficient light source than a 300 W Hg lamp; however, longer reaction times were required to achieve comparable yields with the lower-power light source. These longer residence times further lowered the reactor productivity to 0.15 mmol/h [24].

#### Photooxygenations

Direct oxygenation of organic molecules through the photosensitised addition of singlet oxygen represents an atom-economic method of functionalisation and is employed in the synthesis of a range of compounds of commercial interest including fragrances and pharmaceuticals. One major disadvantage of using this reaction on a large scale is the potential for fires or detonation of accumulating peroxide products. Other disadvantages include inefficient irradiation of bulk solutions, which combined with the extremely short lifetime of singlet oxygen means that lengthy irradiations are often required. These issues can be overcome through the use of continuous-flow chemistry: reactions performed in this manner have only a small amount of oxygenated solvent and peroxide product present at any one time, and this can be reduced immediately upon leaving the reactor. The smaller reactor volumes involved in flow chemistry also mean that irradiation is efficient and hence that the singlet oxygen generated can react within its short lifetime.

**Scheme 7:**
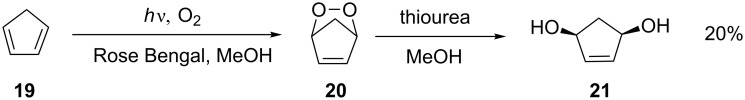
Photooxygenation of cyclopentadiene.

Falling-film microreactors have been employed in the Rose Bengal-sensitised oxygenation of cyclopentadiene **19** (Scheme 7) [30–31]. Although this method allowed good temperature control and the immediate quenching of the potentially explosive peroxide intermediate **20** as it formed, the process was low yielding (20%), and whilst 0.95 g of product **21** was produced the lack of reported details make it difficult to say how scaleable this is likely to be.

**Scheme 8:**
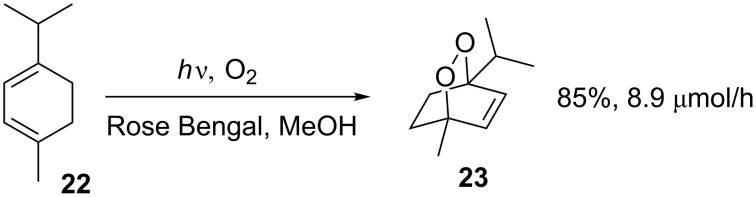
Preparation of the anthelmintic ascaridole **23**.

Rose Bengal has also been employed as a sensitiser in a microchip reactor equipped with a 20 W tungsten lamp for the addition of singlet oxygen to α-terpinene to yield the anthelmintic asaridole (**23**, Scheme 8) [32]. Comparison of this microflow reaction to a batch reaction using a 500 W tungsten lamp showed that although the microflow reaction gave a higher yield (85% versus 67%), the productivity of the flow reactor was markedly lower (1.5 mg/h versus 175 mg/h). This highlights one common issue with moving to microflow photochemistry: although yields may increase, productivity can be significantly lower.

**Scheme 9:**
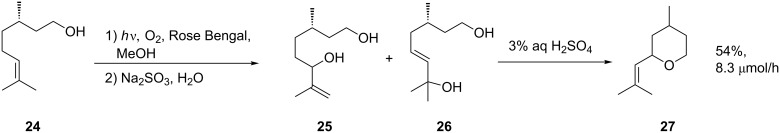
Production of rose oxide **27** from (−)-β-citronellol (**24**).

A glass-loop microreactor was employed in the sensitised oxygenation of (−)-β-citronellol (**24**) shown in Scheme 9, an important reaction for the synthesis of the fragrance rose oxide **27**. It was shown that Rose Bengal was approximately twice as effective a sensitiser as Ru(*t*-bpy)_3_Cl_2_ when a 450 W Xe lamp was employed. Through the use of an LED light source in the microreactor, the authors reported that the microflow system was slightly superior to the batch-type Schlenk reactor in terms of space–time turnovers and photon efficiency; however, the required 400 min irradiation to achieve reasonable conversion on a 1 mmol scale would likely cause difficulty in finding synthetic utility for the reactor [33–34]. It is also interesting to compare the efficiency of the reactor with that of alternatives; despite the reported high photon efficiency, an LCE comparison [35] of six methods for the conversion of β-citronellol into rose oxide showed the above method to be less competitive, mainly due to the long irradiation times causing high consumption of electrical power. It should be noted that an alternative photochemical oxidation using solar radiation was found to be highly efficient, second only to the industrial Dragoco protocol (mercury-arc lamp) [35].

Microflow photochemistry can add a further advantage in photooxygenation reactions through the use of dual-channel reactors. With this type of reactor, oxygen is passed down a second channel, which runs parallel to the channel containing the reaction mixture, and a porous wall allows the diffusion of the oxygen into the reaction mixture. This method avoids the need to oxygenate the solvent before injection, as well as issues such as oxygen depletion in the reaction mixture during the reaction, and bubble formation. Such reactors have been shown to be effective in the oxidative degradations of para-chlorophenol, toluene [36], phenol and methylene blue [37] with a deposited TiO_2_ photocatalyst.

Aside from oxidative degradations, dual-channel reactors have been used for synthetically useful transformations. Returning to the oxidation of α-terpinene, moderate yields of ascaridole were obtained by using a silica-supported fullerene promoter [38]. Similarly, L-methionine was efficiently oxidised to the corresponding sulphoxide in the same reactor [38]. Again, an issue with the reactor was the low productivity: in the case of the oxidation of α-terpinene, productivities were in the order of 10 mg/h, whilst the oxidation of methionine proved less productive at 4.5 mg/h. Neither of these seems likely to be of synthetic use. However, not all dual channel microreactors need suffer such low productivity; the oxidation of β-citronellol (**24**) has been performed in the dual channel reactor designed by Kim et al. and gave a daily output of 45.5 mmol (1.9 mmol/h) by using methylene blue as the sensitiser and a 16 W LED light source, despite the reactor volume being only 285 µL [39]. This output was 2.6 times that of a 50 mL batch reactor. The same reactor was also successfully applied to the oxidation of allylic alcohols for the synthesis of the antimalarial artemisinin, and the conversion of α-terpinene to ascaridole. Addition of a second oxygen-containing channel gave a triple channel reactor that showed even higher space–time yields; however, flow rates are not reported, making it impossible to calculate the productivity of the reactor [20].

Whilst yet to be employed in a synthetic context, it has been shown that porous silica nanoparticles can effectively produce singlet oxygen when irradiated with LEDs in the oxidative degradation of 1,3-diphenylisobenzofuran [40].

#### Photocatalytic reactions

Photocatalytic reactions are an area in which continuous flow can be particularly advantageous due to the large surface-to-volume ratio ensuring efficient irradiation of the whole reaction media; this is especially useful if the photocatalyst is solid supported. The immobilised catalyst of choice is titanium dioxide, both with and without Pt doping, due to its photochemical stability. The photocatalyst can be effectively deposited onto a microreactor surface, thus eliminating the need for subsequent removal of the catalyst in dispersed powder form as would be required in a batch reactor. This methodology has been shown to be effective in the oxidative degradation of a range of organic compounds, including methylene blue, [41–43] *o*-cresol [44], perchloroethylene [45] and 4-chlorophenol [46], mainly with a view to air and water purification [47]. The following section will focus only on synthetic applications of such reactors.

**Scheme 10:**
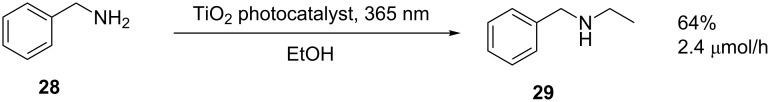
Photocatalytic alkylation of benzylamine.

Photoexcitation of titanium dioxide semiconductors leads to the promotion of an electron to the conduction band, leaving behind a positive hole in the valence band. Thus titanium dioxide can function either as an oxidant by donation of an electron of a reacting molecule into an electron hole, or as a reductant by the donation of an electron in the conduction band of titanium dioxide to another molecule. One synthetic use of titanium dioxide as a photocatalyst is in the alkylation of amines. As shown in Scheme 10, photolysis of a mixture of benzylamine (**28**) and ethanol in the presence of a titanium dioxide photocatalyst gave the ethylamine **29** in high conversion, employing both the oxidative (ethanol to acetaldehyde) and reductive (imine to amine) activity of the photocatalyst [48].

The reaction was also applied to the alkylation of aniline and piperidine. Use of a microsystem conferred a number of advantages over the same reaction under batch conditions: dialkylation could be suppressed as monoalkylated product was removed from the reactor as it formed, avoiding over-reaction; UV-LED light sources could be employed, requiring less power than the lamps typically used for the same reaction under batch conditions; and the reaction could be performed by using Pt-free titanium dioxide, something which had been shown to be unsuccessful under batch conditions. Nevertheless, the low flow rates and low concentrations involved limited output to 2.4 µmol/h, leaving significant questions over its synthetic utility [48–50].

**Scheme 11:**
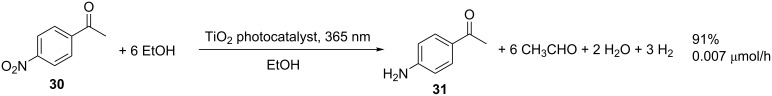
Photocatalytic reduction of 4-nitroacetophenone.

Other redox chemistry to be performed by using titanium dioxide photocatalysts includes the selective reduction of nitro groups to amines in the presence of ketones (Scheme 11) [50–51], and the oxidation of benzaldehyde to benzoic acid, albeit with incredibly poor conversion [52].

**Scheme 12:**
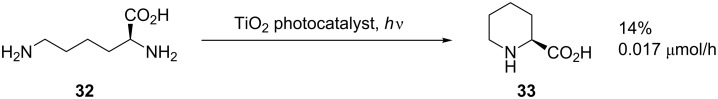
Conversion of L-lysine to L-pipecolinic acid.

The conversion of L-lysine (**32**) to L-pipecolinic acid (**33**, Scheme 12) has also been investigated by using a titanium dioxide photocatalyst. The reaction was found to give poor results under both microflow and batch conditions, both giving a yield of 14%. Although the reaction is stated to have had a significantly faster conversion rate in the microreactor, when the volumes involved are taken into account, output of the batch reaction is reported to be 67 times greater than that of the microflow setup. A further problem with the reaction under both sets of conditions was the erosion of enantiopurity, the ee of the final product being 50% and 47% in microflow and batch reactors, respectively [53].

**Scheme 13:**
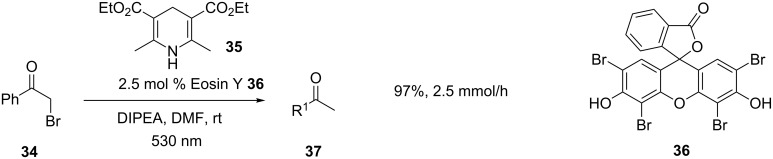
Photocatalytic hydrodehalogenation.

Photocatalysis can also be performed by using visible light. This has been applied to the hydrodehalogenation of α-haloketones by using the dye Eosin Y (**36**) as a photocatalyst, and both DIPEA and Hantzsch ester **35** as electron donors (Scheme 13). The reaction was shown to be high yielding for a number of chlorides and bromides. Again, lower reaction times were reported for the microflow than for the batch reaction, and in this case the productivity of the microflow reactor was shown to be significantly higher than that of the batch reactor (2.5 mmol/h versus 0.4 mmol/h) [54–55].

**Scheme 14:**
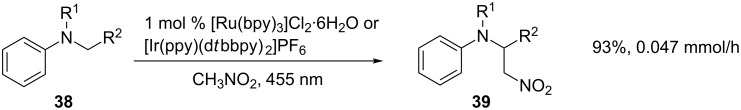
Photocatalytic aza-Henry reactions.

Aza-Henry reactions can also be performed with visible-light photocatalysis, in this case with either ruthenium or iridium catalysts (Scheme 14). Again, residence times in the 100 µL microreactor were significantly shorter than those for the batch reactor, and in this case the microflow system also allowed reactions that were entirely unsuccessful under batch conditions to be conducted [54].

**Scheme 15:**
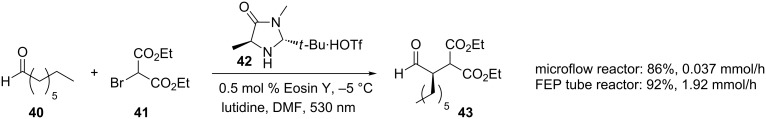
Photocatalytic α-alkylation of aliphatic ketones.

Eosin Y (**36**) catalysis was also applied to an organocatalytic (**42**) photoredox α-alkylation of octanal (**40**, Scheme 15) to aldehyde **43**. The reaction proved to be high yielding under both batch and microflow conditions, and a reduced temperature gave high enantioselectivity. The low productivity of the 100 µL microreactor led the reaction to be transferred to an FEP macroflow reactor for further scale-up, which led to hugely increased productivity. This does, however, demonstrate the utility of microreactors for initial screening of reaction conditions prior to scale-up [54]. Later work demonstrated that the reaction could be extended to a number of aldehydes with good yields and high to excellent ee’s [55].

Photocatalysis in microreactors can also be applied to gas-phase reactions. For instance, the titanium dioxide photocatalysed oxidation of both carbon monoxide and methanol has been followed in a microreactor through the use of quadrupole mass spectrometry (QMS). The levels of time resolution and versatility of detection offered by this method were reported to be far better than that available from conventional GC analysis and, thus, gave data ideally suited to mechanistic studies [56].

The potential of microreactors in the photocatalytic splitting of water has also come under investigation. In this case a rhodium-containing inorganic photocatalyst was employed, and again online QMS permitted mechanistic studies to be performed. Although the results demonstrated the quantum yield for the gas-phase reaction in simulated solar light to be substantially lower than that of the solution-state reaction (0.16% versus 5.5%), it was shown that conversions of up to 43% could be achieved [57].

#### Photodecarboxylation reactions

**Scheme 16:**
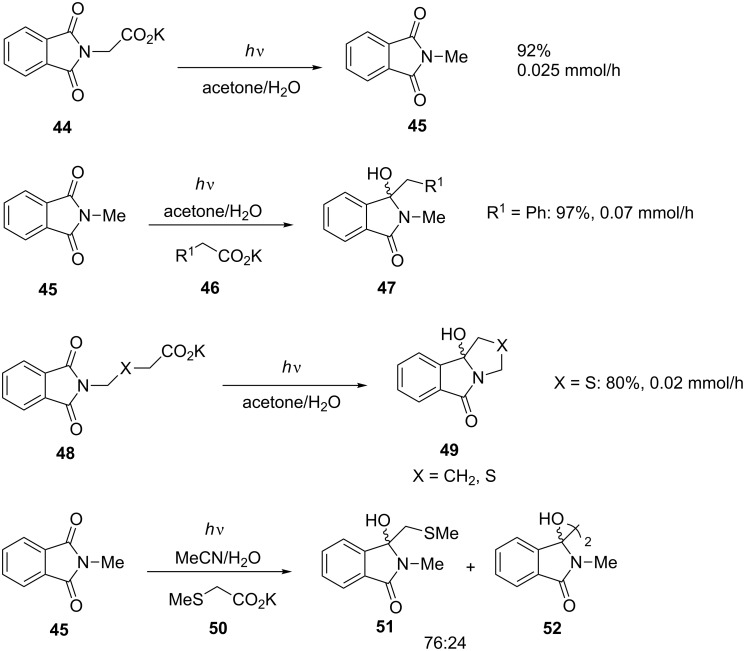
Decarboxylative photochemical additions.

The acetone-sensitised photodecarboxylation chemistry initially developed by Griesbeck [58] under batch conditions was suggested as being ideally suited to microflow conditions [59–60]. The chemistry involves the decarboxylative addition of potassium carboxylates to phthalimides, thus offering an alternative to Grignard reagents that can be employed under aqueous conditions [58]. Initially, the α-photodecarboxylation of phthaloyl glycine **44** (Scheme 16) was investigated in a microflow Dwell device and compared with the reaction under batch conditions. The microflow reactor required a shorter residence time than the Rayonet reactor, which also required 220% more irradiation, but the reactor volumes are too different to make really meaningful comparisons. If productivity is compared, the batch reactor is higher at 0.89 mmol/h compared to 0.025 mmol/h for the microflow setup [61].

Of more synthetic interest is the addition of carboxylates **46** to phthalimides **45** shown in Scheme 16. This reaction has also been compared for microflow and batch reactors for a number of substrates [61–62], and again, although residence times are lower under microflow conditions, productivity is higher for the batch reactor. For instance, with **46** (R^1^ = Ph), productivity is 0.07 mmol/h versus 4.0 mmol/h for the microflow and Rayonet reactors, respectively. When the same light source is used the Rayonet remains the most productive at 0.44 mmol/h [61].

Tethering the potassium carboxylate function to nitrogen (e.g., **48**) allows the decarboxylative additions to be performed as intramolecular cyclisations to the heterocyclic systems **49** (Scheme 16). The reactions of both substrates proved successful in both batch and microflow reactors with the batch reactor proving the most productive (0.54 versus 0.02 mmol/h). When the flow-reactor light source was employed in the batch reactor, a substantial decrease in conversion was observed in the same time period (0.54 versus 0.16 mmol/h). Whilst this remains 7.5 times greater than the flow reaction, the sense of sacrificing conversion for productivity would depend on a number of factors, particularly the value of time and products versus the value of the starting material. However, as the batch reaction using the lower powered light source was not run for an extended time it is impossible to say what the productivity would have been had the reaction been allowed to near completion [61].

In an extension of the above reactions, it was shown that α-thioalkyl-substituted carboxylates **50** could be added to phthalimides **45** in a microflow reactor (Scheme 16). Although the flow reaction proved successful, the final ratio of the desired product **51** to the unwanted reductive dimer **52** was identical to that achieved in a batch reactor [63].

It has been shown that 4,4’dimethoxybenzophenone (DMBP) can be used instead of acetone as a sensitiser in these reactions, thus allowing the use of UVA rather than UVB irradiation. Although this is desirable from a technical point of view, particularly with regard to the use of LED light sources, it does introduce further purification issues. The reactions shown in Scheme 16 were performed in both microflow and batch reactors, and although the microflow reactor was seen, in some circumstances, to be more selective for the desired reaction versus reduction, the productivity of the batch reactor was consistently superior [64].

#### Miscellaneous photochemical reactions

**Scheme 17:**
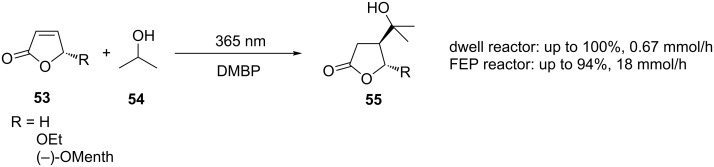
Photochemical addition of isopropanol to furanones.

DMBP has been used as a sensitiser for another reaction performed in a microflow reactor. Oelgemöller et al. showed that the addition of isopropanol (**54**) to furanones **53** (Scheme 17) could be performed in a microchip reactor using an LED light source [65]. The results showed the reaction time to be shorter than for a Rayonet reactor with flow rates of 2.6 µL/min. Further work focussed on the use of a dual microcapillary tower reactor [66] and a Dwell device, both of which gave similarly high conversions but with much improved flow rates (230 µL/min and 340 µL/min, respectively). Both devices approach the productivity regime of the batch reactor with which they were compared (1.4 mmol/h batch versus 0.46 mmol/h tower and 0.67 mmol/h dwell) [67]. This progress was built upon by the manufacture of a ten-capillary device fabricated from FEP tubing (1.6 mm o.d., 0.8 mm i.d.), each tube having an internal volume of 5 mL. The tubing was wrapped around two 18 W UVA (365 nm) lamps, five tubes per lamp, giving a device that clearly fits into the macroflow regime as defined in this review. By making more efficient use of the light, productivities were increased to 1.8 mmol/h per tube. Additionally, it was demonstrated that the ten capillaries could be used either for parallel synthesis or, by performing the same reaction in each capillary, a single product could be formed at up to 18 mmol/h, which is a significant improvement on the microreactor starting point [68].

**Scheme 18:**
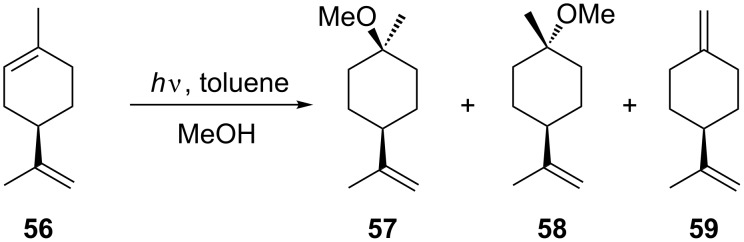
Photochemical addition of methanol to limonene.

Toluene has been employed as a photosensitiser in the addition of methanol to limonene (**56**, Scheme 18). The reaction was performed in a quartz microreactor; however, the reaction suffered from poor product selectivity and d.e. Comparison with a batch reactor showed that the batch reactor gave much higher conversion [50,69]. Subsequent work has shown that the conversion of this reaction in a microflow reactor can be improved through the use of high-power, high-pressure Hg lamps [28].

**Scheme 19:**
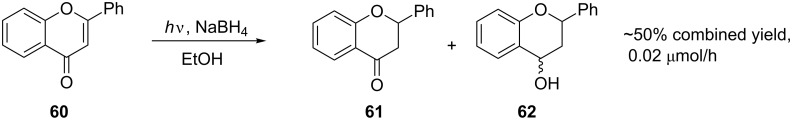
Light-promoted reduction of flavone.

Microflow photochemistry has been applied to the challenging 1,4-reduction of flavones **60** with NaBH_4_ (Scheme 19). It was shown that under photochemical reduction conditions a number of products, including ethyl salicylate and various dimers, were formed. Use of a photochemical flow system rather than a batch reactor was found to give a greater conversion at the expense of increased dimer formation, both effects being ascribed to the greater average photon density in the flow system [70].

**Scheme 20:**
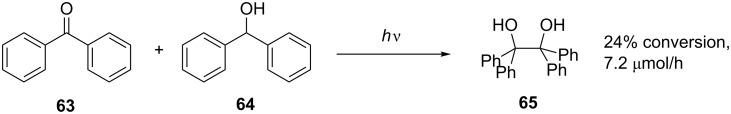
Photoreduction of benzophenone with benzhydrol.

Mechanistic studies of the photoreduction of benzophenone (**63**) with benzhydrol (**64**) (Scheme 20) have been performed in a microflow reactor, allowing the quantum yield of the reaction to be determined by using far less solvent than in standard methods [71].

**Scheme 21:**
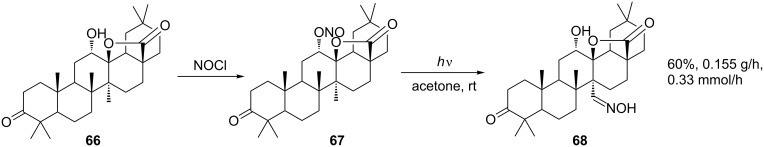
Barton reaction in a microflow system.

A microflow reactor was used in the scaling up of the production of a key intermediate for the endothelin receptor antagonist myriceric acid A (Scheme 21). The reaction was optimised in a single-channel microreactor, which demonstrated that doubling the residence time allowed the switch from a 300 W Hg lamp to a 15 W black light, i.e., a significant increase in photon efficiency, and resulted in an increased percentage yield (71% versus 56%). The same yields could be achieved by using a 1.7 W UV-LED under identical conditions, making the reaction more photon-efficient still. These conditions were then applied to a multichannel microreactor, which allowed the synthesis of multigram quantities of the desired intermediate at rates of up to 0.155 g/h [72–73]. This is an example where the high value of the starting material **66** makes achieving a high percentage yield very important, and although it remains possible that switching from a microflow system to batch reactor would increase productivity at the expense of percentage yield, it is unlikely that this would prove more cost effective.

**Scheme 22:**
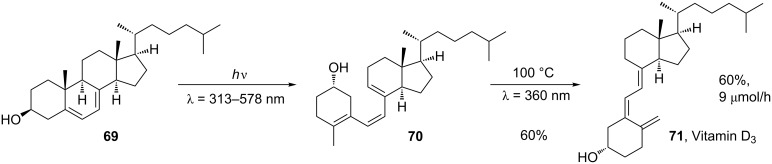
Microflow synthesis of vitamin D_3_.

Microflow photochemistry has also been applied to the synthesis of another steroidal compound, vitamin D_3_ (**71**, Scheme 22). By performing the synthesis in a microreactor, the two stages could be performed consecutively under two different conditions, giving good selectivity for the desired product from a number of common byproducts. Unfortunately, the productivity of the reactor was very low due the optimal flow rate being 5 µL/min [74].

**Scheme 23:**
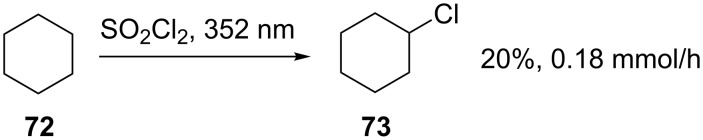
photochemical chlorination of cyclohexane.

**Scheme 24:**
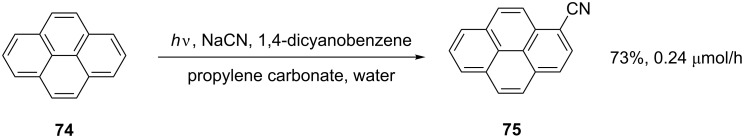
photochemical cyanation of pyrene.

Photocyanation and photochlorination have both been investigated as reactions for microflow systems. Use of a glass dwell device allowed the formation of chlorocyclohexane (**73**) by chlorination of cyclohexane (**72**) with sulfuryl chloride under 15 W black-light irradiation (Scheme 23); however, the reaction was relatively inefficient and productivity was low (0.18 mmol/h) [75]. A biphasic photocyanation of pyrene (**74**, Scheme 24) was shown to be efficient in a polymer microchannel reactor, but the low flow rate led to very low productivity (0.24 µmol/h) [76].

Finally, two studies have demonstrated the utility of coupling online analysis to photomicroreactors, for following reactions and detecting short-lived intermediates. A microflow reactor using photonic crystal fibres was coupled to a mass spectrometer to allow the conversion of cyanobalamin to aquabalamin to be followed. The technique was found to allow much more rapid analysis when compared to a cuvette based approach; however, one issue highlighted was that in general it did not permit quantitative analysis [77]. Another approach to following microphotochemical reactions by online analysis involved UV detection. The formation of benzopinacol from benzophenone was used as a model reaction and quartz reactor construction allowed both the use and detection of shorter wavelengths than would be allowed by Pyrex. The data generated was compared to that generated by HPLC analysis; however, although both sets of data showed the same trend, there were differences in concentration values. This was assumed to be due to continued photoreaction in the HPLC samples (thus leading to inaccurate HPLC data), and indeed use of the online analysis did allow a profile of conversion versus flow rate to be determined [78]. Although at a relatively early stage, both devices show how microflow photochemistry is ideally suited to rapid process optimisation, and it is to be hoped that this potential receives further investigation in the future.

### Macroflow photochemistry

#### Photocycloadditions

The reactor that has served as the prototype for many of the studies summarised in this review of macroflow photochemistry was first reported by Booker-Milburn and Berry in 2005 [21]. In this detailed study, UV-transparent fluorinated ethylene propylene (FEP) tubing (3.1 mm o.d., 2.7 mm i.d.) was used to construct a simple but highly effective single-pass continuous-flow reactor (Figure 3). This reactor demonstrated for the first time how synthetic organic photochemical reactions can be scaled up in a traditional laboratory fume hood to produce multigram quantities of materials without the need for particularly specialist equipment.

**Scheme 25:**
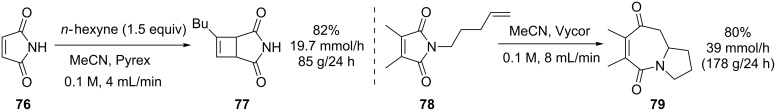
Intermolecular [2 + 2] cycloaddition of maleimide (**76**) and intramolecular [2 + 2] cycloaddition of dimethylmaleimide derivative **78** under flow conditions.

By using an inexpensive peristaltic pump, the [2 + 2] cycloaddition of maleimide (**76**) and *n*-hexyne was run continuously for 24 hours under optimised conditions for a custom-built Pyrex reactor (Scheme 25). This reaction produced 85 g of isolated cyclobutene product **77**. A Vycor reactor, driven by a 600 W lamp gave an 83% conversion when a 0.4 M solution was passed through at 8 mL/min. This corresponds to a productivity of 159 mmol/h, which if run over the same 24 hour period would yield 685 g of product. The higher productivity of the Vycor reactor illustrates the importance of glassware choice for UV transmission. The *N*-pentenyl substituted dimethyl maleimide **78** underwent a [5 + 2] photocycloaddition to the corresponding azepine **79** with a productivity of 39 mmol/h.

**Scheme 26:**
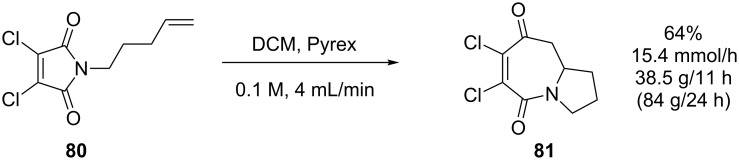
Intramolecular [5 + 2] cycloaddition of maleimide under flow conditions.

The same reactor was also used to optimise the [5 + 2] cycloaddition of *N*-pentenyl-3,4-dichloromaleimide **80** (Scheme 26), a substrate sensitive to over-irradiation due to secondary reactions of the bicyclic azepine product **81**. The scale-up of this reaction was hugely impractical as a batch process. For the maximum tolerated concentration of 0.02 M, the product can only be produced in ~0.5 g amounts, at 50–65% yield, using a conventional batch apparatus. The convenience and superiority of flow photochemistry over batch can be graphically illustrated by the fact that 60 g of **80** was converted to 38.5 grams (64% isolated yield) of **81** in a single 11 h run (0.1 M, 4 mL/min), with recovery of 15.6 g of starting material [79]. To process the same amount in batch at 65% conversion would require 120 *individual* 0.5 g scale reactions with no recovery of starting material.

**Scheme 27:**
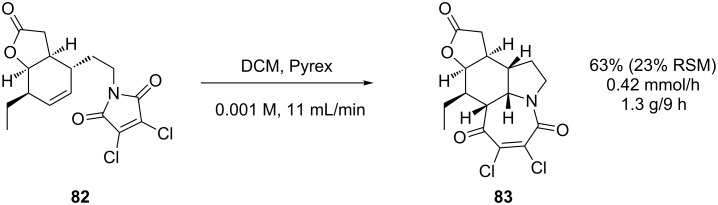
Intramolecular [5 + 2] cycloaddition as a key step in the synthesis of (±)-neostenine.

This FEP reactor was essential for the protecting-group-free synthesis of (±)-neostenine [80]. The key step utilised the [5 + 2] cycloaddition (Scheme 27) for the construction of the pyrrolo[1,2-*a*]azepine core **83**. As a batch process, this particularly sensitive reaction could only be performed on a 50 mg scale giving yields from 40–60% with full consumption of starting material. It would therefore require 42 individual batch reactions to process the 2.1 g of available precursor. When performed under flow conditions, this material was processed in a single 9 hour run with the recovery of 23% starting material **82**.

**Scheme 28:**
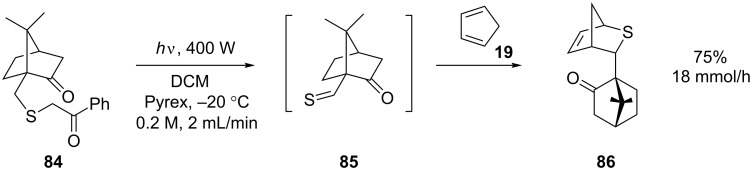
In situ generation of a thioaldehyde by photolysis of a phenacyl sulfide.

Soon after it was first reported, the Booker-Milburn/Berry reactor was utilised by Aggarwal et al. to scale up the photochemical generation of a thioaldehyde **85** (Scheme 28) [81]. The in situ generated species underwent spontaneous Diels–Alder cycloaddition in the presence of cyclopentadiene. The reaction was performed on 18.2 g (60 mmol) of phenacyl sulfide **84** under batch conditions in neat cyclopentadiene to give a 65% yield after 9 hours. Under optimised flow conditions 38 g (126 mmol) of the sulfide was irradiated in DCM (0.2 M) in the presence of 40 equiv cyclopentadiene at 2 mL/min (5.25 hours in total) to give a 75% yield of **86**.

One issue with photochemical flow reactions, which can sometimes be overlooked, is the reduction in performance of the reactor as material is deposited on the tubing walls. This is rarely an issue when the reaction is performed on a small scale/short run time but a thorough evaluation of the performance of a reaction must also take the maximum operational time into account.

**Scheme 29:**
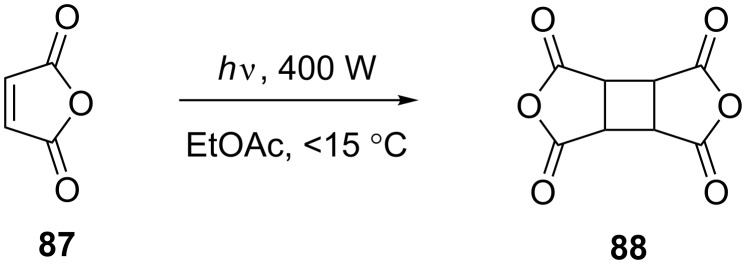
Photodimerisation of maleic anhydride.

The photodimerisation of maleic anhydride (**87**, Scheme 29) is one reaction that poses such a problem since the product **88** is insoluble in common organic solvents. As a batch process the reaction is particularly inefficient since the product suspension scatters light and coats the immersion well. The precipitated product can also pose a problem in a flow reactor as it can adhere to the walls and even block the flow channels completely.

This issue has been addressed in a study by Horie et al. [82]. The FEP tubing of a flow reactor clogged up within an hour under conventional flow conditions with a single solvent. Nitrogen gas was introduced creating a regular stream of bubbles, which broke the solution up into a series of discrete liquid portions in the flow channel. This gas/liquid "slug flow" enabled the precipitated product to be transported more efficiently through the reactor. The individual segments are described as acting like a “micro-batch reactor” containing the mobilised solid. It was found that tubing of i.d. around 1 mm or less was required to produce a regular gas/liquid slug flow. The reactor was also immersed in an ultrasonic bath to further reduce the risk of product adhesion. In this way the reactor could be run continuously for over 16 hours. Compared to the batch process the maleic anhydride dimer **88** was formed in a higher purity since over-irradiation is avoided. A higher overall conversion can be achieved by continuous filtering of the product and recycling of the solution.

**Scheme 30:**
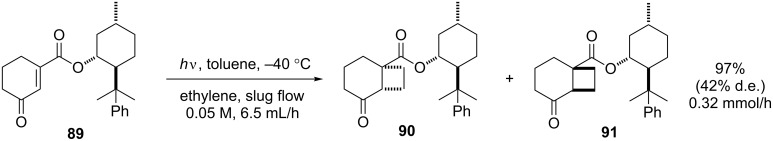
[2 + 2] cycloaddition of a chiral enone with ethylene.

A gas-liquid slug flow was also recently used for the [2 + 2] cycloaddition of a chiral cyclohexenone **89** with ethylene (Scheme 30) [83]. The reactor comprised of FEP tubing (1.0 mm i.d.) wrapped around a quartz immersion well with nine windings. The reaction was driven by a 500 W medium pressure mercury lamp and the solution delivered by a syringe pump. The d.e. of the product was found to be dependent on the reaction temperature and a distinct advantage of the flow reactor is that the temperature can be more precisely controlled than in a batch process. This is achieved by immersing the FEP-wrapped well in a methanol-containing cooling bath. At a given temperature the d.e. of the products **90**/**91** under flow conditions was superior to that when carried out as a batch process.

Flow conditions were employed by Seeberger et al. in the [2 + 2] cycloaddition of maleimide-functionalised poly-L-lysine with alkyne tethered glycol-dendrons to form cyclobutenes [84]. The reactions were performed on a sub-millimolar scale, but the precise control over reaction conditions with the flow apparatus allowed for high yields of the complex dendronic products.

**Scheme 31:**
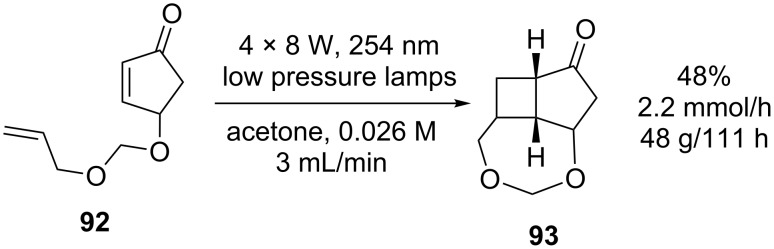
Intramolecular [2 + 2] cycloaddition of a cyclopentenone.

Nettekoven et al. [85] trialled a continuous flow reactor consisting of an Ehrfeld Photoreactor XL driven by a custom built bank of four 8 W low-pressure lamps. The intramolecular cycloaddition of cyclopentenone **92** (Scheme 31) was optimised by varying the reactor channel thickness (20–90 μm), concentration and flow rate. Under optimised conditions 100 g of starting material in acetone (0.026 M) was irradiated at a flow rate of 3.0 mL/min to give **93** in an isolated yield of 48%. The reactor was flushed through with methanol every 24 hours to remove deposits of polymeric side-products, which can reduce the yield of the reaction.

#### Isomerisations and rearrangements

**Scheme 32:**
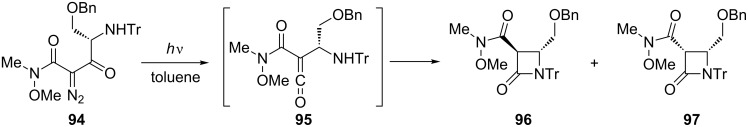
Photochemical Wolff rearrangement and cyclisation to β-lactams.

The irradiation of diazo compounds often leads to the loss of molecular nitrogen along with the formation of a highly reactive carbene species. The carbene generated from the photolysis of α-diazo-β-ketoamide **94** (Scheme 32) underwent a Wolff-rearrangement to ketene **95**, which cyclized to β-lactam diastereomers **96** and **97** [86]. As a batch process the irradiation was performed by irradiating a cooled toluene solution (1.1 mmol, 0.01 M) of the diazo compound in a Pyrex flask with an external medium-pressure mercury lamp. After 7 hours an overall yield of 90% was obtained (0.14 mmol/h).

In order to increase the productivity and scalability of the reaction the cooled solution (1.82 mmol, 0.01 M) was continuously circulated around the lamp through a coil of FEP tubing (3.2 mm o.d., 1.6 mm i.d.) with a total volume of 15 mL. The flow rate was set to 12.5 mL/min although this was of course not a single-pass operation. This setup gave an overall yield of 81% after just 3.5 hours (0.42 mmol/h), a productivity which could probably be improved by wrapping the FEP tubing around the full length of the lamp.

As a safer alternative to the medium-pressure lamp a 100 W CFL was used to drive the reaction. When used in an analogous flow configuration, the solution (1.82 mmol, 0.01 M) was circulated at 5 mL/min for 48 hours to give an overall yield of 91% (0.035 mmol/h). The corresponding batch reaction required 18 hours to process just 0.18 mmol of the diazo compound in a 95% yield (0.01 mmol/h).

**Scheme 33:**
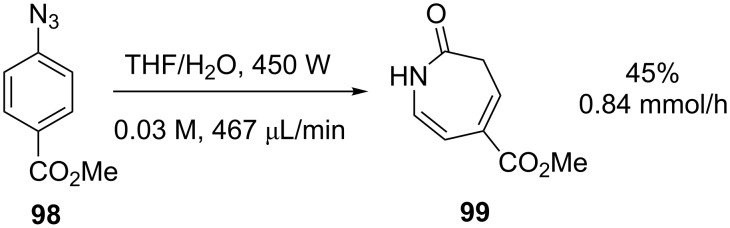
Photochemical rearrangement of aryl azides.

The photolysis of aryl azides in the presence of water provides an easy access to the 3*H*-azepinone ring system. Unfortunately, the reaction suffers from the need for extensive irradiation times and is prone to the formation of byproducts and product decomposition. As a batch process, the photolysis of 2.0 g of methyl 4-azidobenzoate (**98**, Scheme 33) in THF and water (0.05 M) had previously been reported to give a 45% yield in 20 hours, representing a productivity of 0.25 mmol/h [87]. Seeberger and workers have since revisited the reaction and, through the use of a continuous flow reactor, obtained **99** with a productivity of 0.84 mmol/h when irradiating the same substrate [88]. Flow conditions allowed for the precise control over the reaction time necessary to optimise this sensitive reaction.

**Scheme 34:**
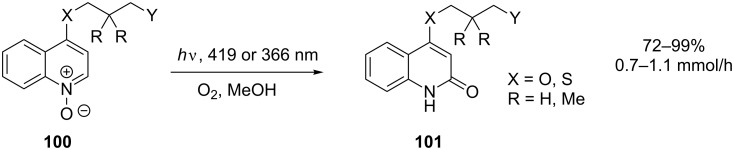
Rearrangement of quinoline *N*-oxides to quinolones.

Photochemical rearrangements of the *N*-oxide moiety represent another important class of reactions that are able to transform an aromatic ring [89]. When synthesising a range of 4-substituted quinolone **101** derivatives from quinoline *N*-oxides **100** (Scheme 34), Bach and co-workers found that the yield was reduced as a result of [2 + 2] dimerisation of the photochemically active product [90]. This was prevented by performing the reaction at reduced concentration (6–7 mM) using fluorescent UVA lamps (419 and 366 nm) in the presence of oxygen as a triplet quencher. In order to efficiently irradiate the large volumes of dilute reactant solution required, the reaction was carried out as a flow process. A flow reactor was assembled consisting of a 7 mm Duran tube, double coiled with an outer diameter of 75 mm and a height of 200 mm. This was positioned in the centre of a Rayonet reactor with 16 lamps of the required wavelength.

The reactor was also used to produce quinolones bearing tethered alkenes at the 4-position for subsequent intramolecular [2 + 2] cycloaddition [91]. This reaction was completely suppressed during the singlet-mediated *N*-oxide rearrangement by the presence of triplet-quenching oxygen.

**Scheme 35:**
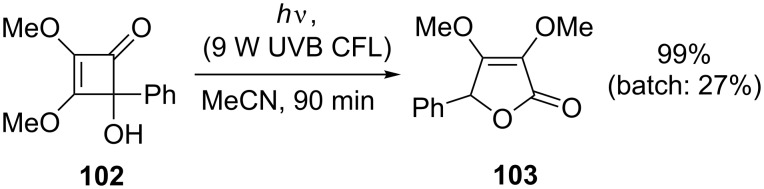
Photochemical rearrangement of cyclobutenones.

Another reaction that has recently been reinvestigated by using a continuous-flow process is the photochemical rearrangement of arylcyclobutenone **102** to 5*H*-furanone **103** (Scheme 35). The original report described a batch reaction utilising a quartz well and a 400 W medium-pressure mercury lamp [92]. A 27% yield of phenyl substituted furanone **103** was obtained after 4 hours, and it was noted that the products were unstable under the photolysis conditions. Harrowven and co-workers constructed a photochemical flow reactor by wrapping PFA tubing (1.6 mm o.d., 1.0 mm i.d.) around a quartz tube [93]. Driving the reaction from within this tube was a compact 9 W PL-S lamp, available with a UVA- or UVB-emitting phosphor in addition to the uncoated 254 nm lamp. The use of the UVB lamp allowed for selective irradiation of the cyclobutenone over the product, thus minimising secondary reactions. The resourceful choice of light source combined with fine control over irradiation time allowed for near quantitative yields to be obtained for a range of derivatives in this useful but previously capricious reaction.

**Scheme 36:**
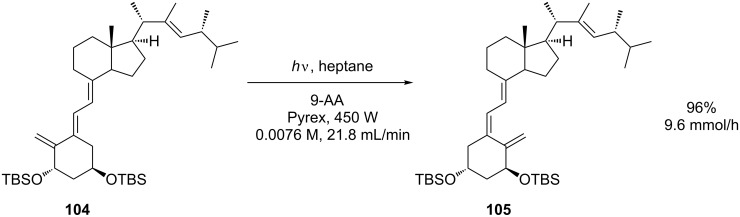
Photoisomerisation en route to a vitamin-D derivative.

As part of a new route to the therapeutic vitamin D derivative doxercalciferol, the sensitized photoisomerisation of **104** to **105** was investigated (Scheme 36) [94]. A continuous-flow photochemical reactor was constructed by using the Booker-Milburn/Berry configuration in the hope that flow conditions would overcome the inherent difficulties in scaling up a batch process. A single layer of tightly coiled FEP tubing (3.18 mm o.d., 1.59 mm i.d.) was wrapped around a cooled immersion well with a Pyrex filter and the reaction was driven by a 450 W MP lamp. In order to optimise the reaction a full factorial design-of-experiments (DoE) study was performed with temperature, concentration, flow rate, and 9-acetylanthracene loading as factors. Optimal conditions correspond to low concentration (5.0 mg/mL) and high flow rate. The reaction temperature and sensitiser loadings were not found to be significant factors over the range investigated in the design space.

#### Photooxidations

As highlighted in the microflow section the in situ generation of singlet oxygen (^1^O_2_) by sensitisers is in principle a simple and environmentally benign method to produce such a reactive reagent. Whilst the synthetic potential for ^1^O_2_ has been demonstrated extensively, several issues with scale-up have prevented its widespread industrial use. As with all photochemical reactions, increasing the scale of a batch reaction by increasing reactor volume alone has an adverse effect on the efficiency. This is a result of the majority of photons being absorbed a short distance from the lamp and is exacerbated by the intense absorption of many photosensitisers.

A widely used solution to this problem is to recirculate solution from a reservoir through an annular reactor. In a particularly novel example, researchers generated ^1^O_2_ on nanoporous silicon excited by the emissions from green LEDs [40]. The reactor was used to decompose diphenylisobenzofuran with an estimated quantum yield of 34% although it was not put to any synthetic use. The scale-up of ^1^O_2_ reactions is also complicated by the need for efficient oxygen delivery to the system. A reservoir of oxygenated solvent poses a considerable safety risk on any scale and a far safer option is to introduce the gas as and when required. This technique, however, can be limited by the low mass transfer of oxygen into many solvents. The use of supercritical carbon dioxide as a solvent in a single-pass reactor enabled the oxidation of citronellol at a rate of 0.27 mmol/min [95]. This method exploited the high solubility of O_2_ in scCO_2_, along with the low viscosity of the fluid, to overcome the mass-transfer issues. The negligible environmental impact of scCO_2_ along with its nonflammable nature could see its use in a particular industrial setting, but the equipment is likely far too specialised to be taken up by many.

**Scheme 37:**
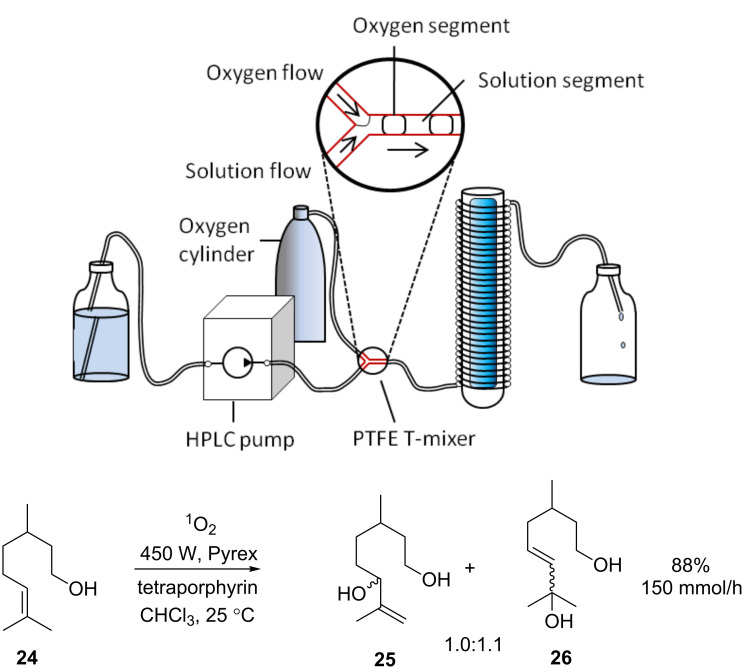
Schematic of the Seeberger photooxygenation apparatus and sensitised photooxygenation of citronellol.

Fortunately, Seeberger and co-workers have recently shown that sufficient mass transfer of O_2_ can be achieved with more conventional equipment if slug flow conditions are employed [96]. A solution of citronellol was mixed with oxygen gas in a PTFE T-mixer before entry into a Booker-Milburn/Berry type FEP flow reactor (1.59 mm o.d., 0.76 mm i.d.) (Scheme 37). The gaseous segments of the biphasic mixture enabled a huge surface area of the solution to be exposed to oxygen. The concentration of oxygen in solution was further increased by increasing the pressure with a 6.9 bar back-pressure regulator. Under optimised conditions near quantitative yields were obtained with a productivity of 2.5 mmol/min. This simple but hugely effective reactor configuration addresses all major issues of sensitised photooxygenation reactions: safe, controlled introduction of oxygen to the solution, and efficient irradiation from the light source.

**Scheme 38:**
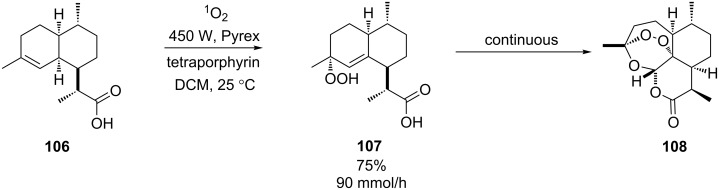
Sensitised photooxygenation of dihydroartemisinic acid.

The synthetic use of the above reactor was demonstrated dramatically in the synthesis of artemisinin **108** [97]. This first-line antimalaria drug was produced as a continuous-flow process from dihydroartemisinic acid in three consecutive steps. TPP sensitised photooxidation of **106** produced the allylic hydroperoxide at a rate of 1.5 mmol/min in 75% yield (Scheme 38). This was followed by acid-catalysed Hock cleavage and triplet-oxygen (^3^O_2_) oxidation. The resulting compound underwent a series of spontaneous condensations to give artemisinin in 45% yield. This is a highlight for flow chemistry in general and demonstrates what can be achieved by the marriage of chemistry with technology.

#### Photocatalytic reactions

**Scheme 39:**
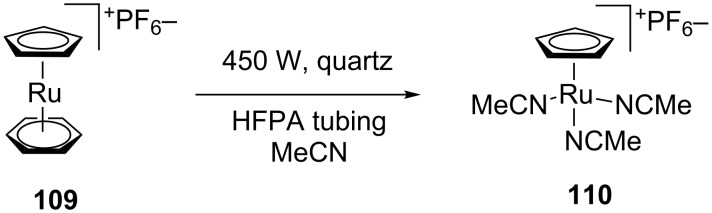
Photochemical preparation of CpRu(MeCN)_3_PF_6_.

As with photooxidations, the widespread use of reactions involving photoactivated catalysts has been marred by the inability to scale up batch processes. The highly reactive cyclopentadienylruthenium complex **110** can be prepared photochemically from the corresponding benzene sandwich complex **109** in near quantitative yield (Scheme 39). When performed as a batch reaction the concentration was limited to 0.02 M and irradiation times in excess of 12 hours were required. A continuous-flow reactor was constructed by wrapping high-purity perfluoro alkoxy alkane (HPFA) tubing (1.58 mm o.d., 0.79 mm i.d.) around a quartz immersion well [98]. This reactor enabled catalyst **110** to be produced at a rate of 1.56 g/h when a 0.06 M solution was pumped at 1 mL/min. The high purity PFA was required since standard PFA tubing leeched plasticiser into solution and reduced conversion.

**Scheme 40:**
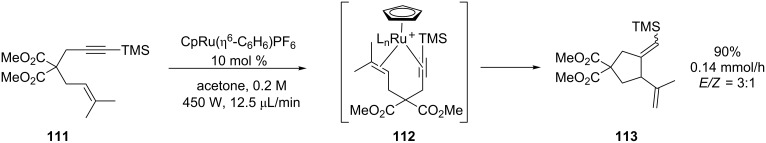
In situ photochemical generation and reaction of a [CpRu]^+^ catalyst.

It was later shown that a catalytically active [CpRu]^+^ species could be generated in situ by direct photolysis of the aryl complex and intercepted by reactants [99]. This avoided the need to prepare and isolate the tris(acetonitrile) complex. The reactivity of the photoactivated species was first demonstrated with an intramolecular ene–yne cycloisomerisation (Scheme 40). No product was observed under batch conditions but the optimised flow conditions gave complete conversion within very short residence times. The reactor consisted of a single coil of quartz tubing (3.18 mm o.d., 0.73 mm i.d.) positioned around a water cooled 450 W MP lamp, with or without a Pyrex filter. Although the reported isolated yield of 90% was obtained with flow rate of 12.5 μL/min, 98% conversion was observed at flow rates up to 125 μL/min through a Pyrex filter and 333 μL/min in the absence of a filter.

**Scheme 41:**
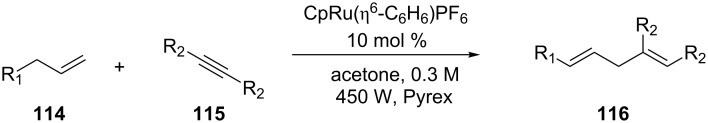
Intermolecular alkene–alkyne coupling with photogenerated catalyst.

The intermolecular alkene–alkyne coupling was also successful for a range of substrates (Scheme 41). Whilst the productivity of the reaction for many of the isolated products appears rather modest (<1 mmol/h) given the power of the lamp, the reactor consisted of a single loop of quartz tubing with internal volume 250 μL. A modified setup that captures the full emissions of the 450 W mercury lamp would likely improve the productivity rate.

**Scheme 42:**
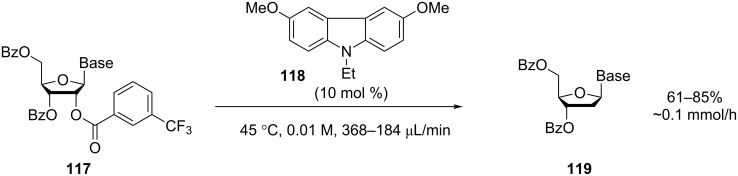
PET deoxygenation of nucleosides.

A more efficient development of the quartz tubing reactor has since been reported by the same group [100]. The reactor features a coil of quartz tubing (3.18 mm o.d., 1.0 mm i.d.) with multiple turns so as to span almost half the length of the 450 W MP lamp. The loss of UV by transmission through the wall was minimised through the use of a highly reflective aluminium mirrored cylinder. Although quartz is superior in terms of transparency and is chemically inert, the fabrication constraints may cause limitations. The reactor was used for a catalysed (**118**) photoinduced-electron-transfer (PET) deoxygenation reaction to produce 2‘-deoxy and 2‘,3‘-dideoxynucleosides. The 2‘-deoxynucleosides **119** were typically produced in yields of around 80% with productivities around 0.1 mmol/h (Scheme 42).

**Scheme 43:**
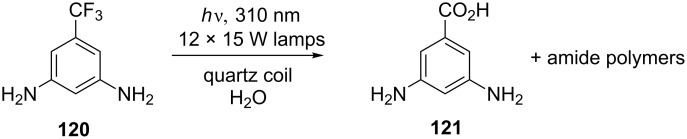
Photochemical defluorination of DABFT.

An earlier example of a quartz-tubing photochemical reactor utilised a squared coil of tubing (5.0 mm o.d., 1.5 mm i.d.) 7 cm wide and 23.5 cm high [101]. The thickness of the tubing walls was compensated for by the impressive array of 15 W LP lamps: six inside the coil and another six external. The reactor was used for the photochemical defluorination of 3,5-diamino-trifluoromethylbenzene (DABFT) **120** in water to give 3,5-diaminobenzoic acid (**121**, Scheme 43). Complete conversion of DABFT was observed at the highest flow rate and concentration tested (1 g/L, 1 mL/min = 0.34 mmol/h). The reaction was not optimised, however, and was not put to any synthetic use.

The above examples have relied heavily on the UV light emitted by medium-pressure mercury lamps. Exposure of the products to the high-energy photons can cause unwanted side reactions. In recent years a vast amount of progress has been made in the field of visible-light-activated photocatalysts. Although central to the discovery of the new reactions, the batch apparatus used has often been ineffective in allowing scale-up to synthetically useful quantities. This is once again due to the strong absorption of the photocatalysts preventing the penetration of light into the bulk of the solution. The first examples of continuous-flow processes being used to carry out such visible-light-mediated photocatalytic reactions in the field of organic synthesis have only just emerged.

**Scheme 44:**
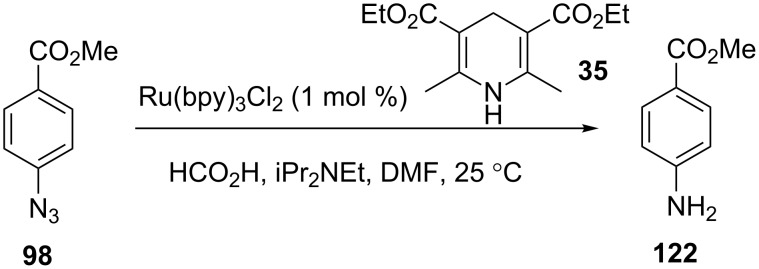
Aromatic azide reduction by visible-light-mediated photocatalysis.

Seeberger and co-workers constructed a reactor with a 4.7 mL volume by wrapping FEP tubing (1.59 mm o.d., 0.76 mm i.d.) around two parallel metal rods held apart so as to form a planar surface [102]. The tubing was positioned between two 17 W white LED lamps, a configuration that efficiently captures the light from the planar light sources. A range of Ru(bpy)_3_Cl_2_ catalysed reactions were trialled and compared to their batch counterparts. The productivity of the flow processes were consistently higher than the previously reported batch results, tolerated lower catalyst loadings and proceeded well in the absence of Hanzsch ester **35**. For example, the reduction of methyl 4-azidobenzoate (**98**) gave **122** in 89% yield at a flow rate of 2.36 mL/min and concentration of 0.1 M in the presence of 1.2 equiv of a Hantzsch ester (Scheme 44). This corresponds to a productivity of over 12 mmol/h. The reaction also proceeded in the absence of the Hantzsch ester, but the flow rate was reduced to 236 μL/min giving a productivity of 1.2 mmol/h.

**Scheme 45:**
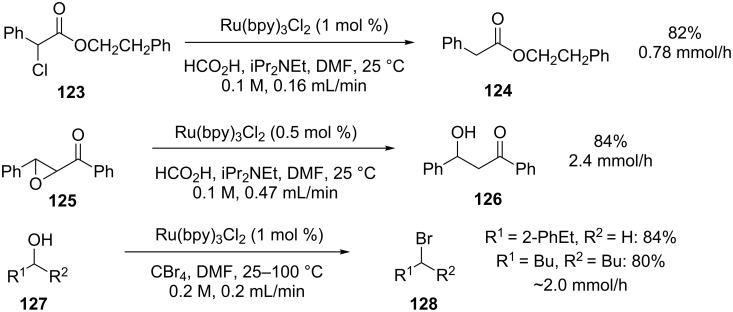
Examples of visible-light-mediated reactions.

Similar productivities [102] and yields were also observed for the reductive dechlorination, reductive epoxide opening and alcohol bromination all in the absence of the Hantzsch ester (Scheme 45). In the case of the bromination, the solution was passed through the photochemical reactor at 25 °C before flowing directly through a PTFE reactor at 100 °C to drive the bromination to completion.

**Scheme 46:**
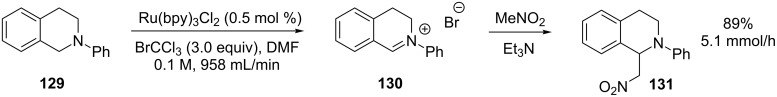
Visible-light-mediated formation of iminium ions.

A similar reactor design was reported soon after by Stephenson et al. [103]. This was constructed from PFA tubing (0.76 mm i.d.) wrapped in figures of eight around two glass tubes so as to capture the light from an assembly of seven blue (447 nm) LEDs operating at 5.88 W. The internal volume of the reactor was 497 μL and a silver mirrored flask was used as a reflector behind the tubing. Impressive productivities were observed when trapping oxidatively generated iminium ions with a range of nucleophiles. For example, a solution of *N*-phenyltetrahydroisoquinoline **129**, BrCCl_3_ and Ru(bpy)_3_Cl_2_ in DMF was passed through the reactor at a rate of 5.75 mmol/h (Scheme 46). This was sufficient for full conversion to the iminium salt **130**, which was reacted immediately with a nucleophile present in the dark receiving flask. In addition to the nitromethane adduct **131**, sodium cyanide, an allylic silane, and an acetylene acted as nucleophiles giving the trapped products in yields of over 80%. When performed on batch, the nitromethane adduct was produced at a rate of just 0.081 mmol/h.

**Scheme 47:**
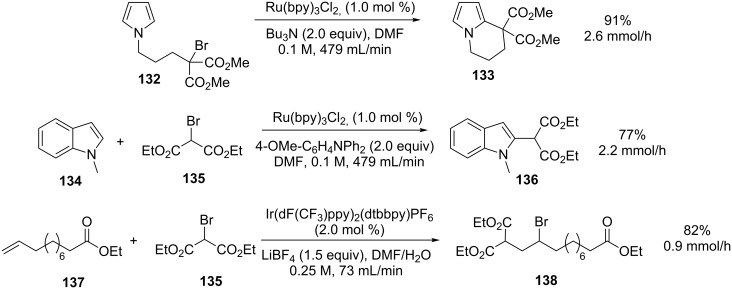
Examples of visible-light-mediated photocatalytic reactions.

Examples are also given for additional reactions that showed a great improvement in productivity when performed with the flow reactor compared to the batch conditions used during initial studies. These included intramolecular radical cyclisations, intermolecular radical indole functionalisations, and intermolecular atom-transfer radical additions (ATRA) using an iridium catalyst (Scheme 47).

**Scheme 48:**
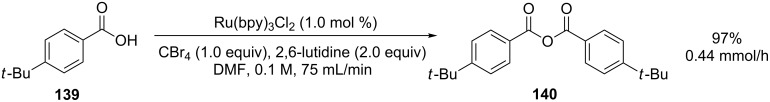
Anhydride formation from a visible-light-mediated process.

The same reactor was also used to demonstrate a new method for the synthesis of symmetric anhydrides through the light-mediated generation of the Vilsmeier reagent by using Ru(bpy)_3_^2+^ and CBr_4_ in DMF (Scheme 48) [104].

**Scheme 49:**
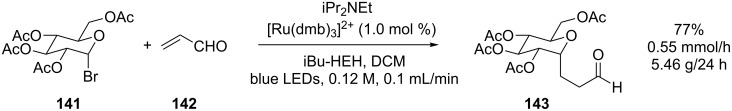
Light-mediated conjugate addition of glycosyl bromide **141** to acrolein.

Another continuous-flow visible-light reactor was also reported at the same time by Gagné and co-workers [105]. The light-mediated conjugate addition of glycosyl radicals to acrolein **142** (Scheme 49) was high yielding on a small scale in batch (0.06 mmol in 1 h, 70%), but it could not be scaled up effectively without extensive irradiation times (2.43 mmol in 24 h, 85%). This issue was solved by conducting the reaction under continuous-flow conditions. The reactor used consisted of FEP tubing (1.59 mm i.d.) conveniently wrapped around a standard Liebig condenser containing three strips of blue LEDs. Two of these were connected in series to give the alkylated glycoside **143** with a productivity of 0.55 mmol/h. The reactor was run continuously for 24 h to yield 5.46 g of this key intermediate for further studies. This example serves to illustrate how difficult photochemistry can be rendered useful in flow by using a well thought out, but simple, reactor design that utilises common laboratory equipment.

**Scheme 50:**
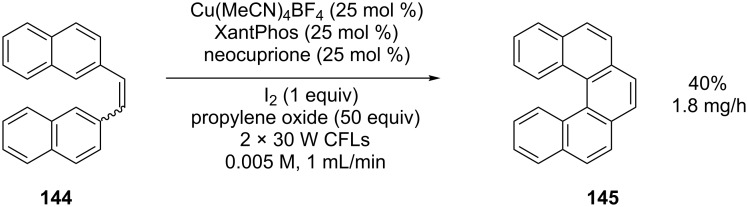
Visible-light-mediated photocyclisation to [5]helicene.

A recently reported example of a visible-light-mediated photocatalytic process utilising flow conditions involved the cyclisation of stilbene derivative **144** to [5]helicene (Scheme 50) [106]. A reactor was constructed by wrapping FEP tubing (2.0 mm o.d., 1.0 mm i.d.) around two 30 W CFLs. Under optimised reaction conditions in a batch apparatus, a 57% yield of [5]helicene was obtained after 120 hours irradiation with one 30 W CFL. A solution of identical concentration and scale was irradiated by using the flow reactor to give the product in 40% yield after just 10 hours. The reaction mixture still had to be passed through the reactor 20 times to obtain this conversion but the flow apparatus clearly allows more efficient irradiation.

## Conclusions

Flow photochemistry has developed rapidly since the early reports just over 10 years ago. Initial studies focussed on the microflow regime, which itself was born out of the “lab-on-a-chip” arena. Since then there have been many reports of various well engineered microflow photochemical reactors. Most of these have shown that many photochemical reactions can be carried out with higher yields (space/time) and selectivities and with fewer side reactions than comparable batch reactors. On the whole, however, microreactors are uncompetitive with classic immersion-well batch reactors when it comes to the key issue of productivity. This is unsurprising given the very low reaction volumes and flow rates involved, and as such comparison of two such different reactor topologies is not useful. Microflow reactors are particularly well placed to make best use of the current developments in LED technology. As microflow reactors cannot make use of a large photon flux, much of the radiation from powerful UV lamps is wasted. Use of arrays of compact LEDs is much more suitable and efficient. At the moment LEDs of λ < 365 nm are expensive, prohibitively so at wavelengths of 300 nm and below where a single LED can cost as much €300. This price will come down in future, but it is likely that only a microflow reactor could benefit from this. With further developments photochemical microflow reactors are likely to find many applications, particularly if they can be coupled with automation: screening for new photoreactions, reaction and wavelength optimisation, drug discovery, micro-actinometers for quantum yield measurements, etc.

Since its introduction in 2005, the FEP macroflow reactor of Booker-Milburn and Berry has demonstrated that batch-locked reactions can be scaled up from sub-gram quantities to over 500 g per day in a single pass. A flagship example of this was recently reported by Seeberger and Lévesque in their continuous (>200 g per day) synthesis of artemisinin, the current front-line treatment for malaria. Related designs have very recently demonstrated that photocatalysis can be carried out in macroflow devices with high productivities. This is a very significant development as photocatalysis is a powerful emerging area for synthetic chemistry and promises to have wide application. The value of FEP and related tube designs lies in the simplicity of their construction: all the tubing, glassware, lamps and pumps are commercially available at a very economical price and a functioning reactor can be set up in a matter of hours in a standard fume hood.

With now easy access to flow photochemistry we hope that the synthetic community at large will make more use of photochemical bond-forming reactions and apply them to their general synthetic problems. As way of stimulus, the following provocative question can be asked: can your ground-state chemistry give you easy, clean and reagentless access to 100 g quantities of molecules with high structural complexity? Flow photochemistry can.
